# The Impact of Virtual Consultations on the Quality of Primary Care: Systematic Review

**DOI:** 10.2196/48920

**Published:** 2023-08-30

**Authors:** Kate Campbell, Geva Greenfield, Edmond Li, Niki O'Brien, Benedict Hayhoe, Thomas Beaney, Azeem Majeed, Ana Luísa Neves

**Affiliations:** 1 Department of Primary Care and Public Health Imperial College London London United Kingdom; 2 Institute of Global Health Innovation Department of Surgery and Cancer Imperial College London London United Kingdom

**Keywords:** remote consultations, primary care, telemedicine, systematic review, teleconsult, quality care, efficiency, socioeconomic status

## Abstract

**Background:**

The adoption of virtual consultations, catalyzed by the COVID-19 pandemic, has transformed the delivery of primary care services. Owing to their rapid global proliferation, there is a need to comprehensively evaluate the impact of virtual consultations on all aspects of care quality.

**Objective:**

This study aims to evaluate the impact of virtual consultations on the quality of primary care.

**Methods:**

A total of 6 databases were searched. Studies that evaluated the impact of virtual consultations, for any disease, were included. Title and abstract screening and full-text screening were performed by 2 pairs of investigators. Risk of bias was assessed using the Mixed Methods Appraisal Tool. A narrative synthesis of the results was performed.

**Results:**

In total, 30 studies (5,469,333 participants) were included in this review. Our findings suggest that virtual consultations are equally effective to or more effective than face-to-face care for the management of certain conditions, including mental illness, excessive smoking, and alcohol consumption. Overall, 4 studies indicated positive impacts on some aspects of patient-centeredness; however, a negative impact was noted on patients’ perceived autonomy support (ie, the degree to which people perceive those in positions of authority to be autonomy supportive). Virtual consultations may reduce waiting times, lower patient costs, and reduce rates of follow-up in secondary and tertiary care settings. Evidence for the impact on clinical safety is extremely limited. Evidence regarding equity was considerably mixed. Overall, it appears that virtual care is more likely to be used by younger, female patients, with disparities among other subgroups depending on contextual factors.

**Conclusions:**

Our systematic review demonstrated that virtual consultations may be as effective as face-to-face care and have a potentially positive impact on the efficiency and timeliness of care; however, there is a considerable lack of evidence on the impacts on patient safety, equity, and patient-centeredness, highlighting areas where future research efforts should be devoted. Capitalizing on real-world data, as well as clinical trials, is crucial to ensure that the use of virtual consultations is tailored according to patient needs and is inclusive of the intended end users. Data collection methods that are bespoke to the primary care context and account for patient characteristics are necessary to generate a stronger evidence base to inform future virtual care policies.

## Introduction

### Background

The onset of the COVID-19 pandemic in 2020 resulted in the rapid expansion of virtual consultations in primary care [1]. The shift from a primarily face-to-face (F2F) model of health care provision led to approximately 70% [2] and 65% [3] of primary care contacts happening virtually in the United Kingdom and United States, respectively.

Virtual consultations can be defined as real-time communication between patients and clinicians through telephone or videoconferencing [4]. It is argued that not only have virtual consultations helped minimize COVID-19 transmission, but they may also improve the efficiency of and access to care [5]. This may be particularly important in rural areas with geographical disparities in service provision [6] and resource-constrained settings with workforce shortages [4]. In addition, virtual care may improve patient-provider communication and engagement [7] and better facilitate multiperson involvement in care [8].

However, concerns have been raised over the speed at which virtual consultations were implemented, with both patients and clinicians reporting lack of confidence in the underlying technology and poorer clinical decision-making as key issues. [9]. Patients have expressed concerns regarding confidentiality, privacy, and the safety of their data [7,8]. Virtual care limits clinicians’ capacity to conduct physical examinations [10] and increases reliance on patients’ abilities to articulate their symptoms [5], posing potential safety risks. Moreover, those with limited access to technology or with lower digital literacy may be at risk of “digital exclusion” [11].

Previous reviews have investigated the impact of virtual consultations on the effectiveness [3,12-15] and efficiency [16,17] of care, often focusing on specific clinical conditions or taking broad definitions of remote services. However, there is a notable lack of systematic reviews assessing the impacts on safety, patient-centeredness, timeliness, and equity, with much of the existing literature limited to scoping or rapid reviews of the evidence [18-21]. Furthermore, although some systematic reviews investigated the impact of virtual care on aspects of quality exclusively in primary care settings [13,16,17,22], others examined heterogeneous evidence, including data on interventions delivered in secondary care facilities or by specialists [3,15]. There is, therefore, a need to comprehensively evaluate the impact on all aspects of care quality specifically in primary care settings.

### Aim of This Review

The aim of this review was to systematically evaluate the impact of virtual consultations on the quality of primary care. We chose to use the Institute of Medicine (IOM)’s theoretical framework to map the impact across 6 domains of quality, including efficiency, effectiveness, safety, patient-centeredness, timeliness, and equity [23]. This widely accepted model for describing care quality has been previously used in other systematic reviews investigating the effects of digital health interventions on the quality of care [24,25]. When evaluating the impact of virtual consultations on quality, it is essential that all quality domains are assessed, as what is beneficial in one domain may be detrimental in another. Therefore, the IOM’s quality of care framework provides a comprehensive model for evaluating the impact of virtual consultations across all aspects of care quality.

## Methods

This systematic review followed the PRISMA (Preferred Reporting Items for Systematic Reviews and Meta-Analyses) checklist [26]. The study protocol was registered with the International Prospective Register of Systematic Reviews (CRD42022362380).

### Search Strategy

Six databases (MEDLINE, Embase, HMIC, PsycINFO, CINAHL, and Cochrane) were searched on June 20, 2022. The search included studies published between January 2017 and June 2022, as the last 5 years have seen most of the shift toward virtual care. Concepts covered in the search strategy primarily included (1) virtual consultations, (2) primary care, and (3) the IOM’s 6 domains of care. The concepts of virtual consultations and primary care were intentionally kept broad to address variations in language, and search terms for the domains of quality were adapted from a previous review [24].

A detailed breakdown of the exact combination of free text and Medical Subject Headings terms used can be found in Multimedia Appendix 1. The reference lists of relevant systematic reviews and gray literature sources were also screened.

### Study Selection Criteria

Studies were included if they focused on adult patients accessing primary care services (including care homes), involved telephone or videoconference consultations delivered by health care professionals, compared outcomes with F2F consultations, and reported outcomes that fit under any of the IOM’s quality of care domains. Detailed descriptions of the inclusion and exclusion criteria are provided in Textboxes 1 and 2, respectively. Studies focusing on specific health conditions were not excluded to characterize the general use of virtual consultations in primary care.

**Inclusion criteria.**

**Population (and setting)**

- Adult patients (mean age ≥18 y) with any health condition accessing primary care services in any geographical location

**Intervention**

- Two-way, synchronous patient-provider virtual consultations delivered via telephone or videoconference by primary care health care professionals or multicomponent interventions involving synchronous remote consultations

**Comparator**

- Consultation delivered face-to-face or the outcomes assessed indicate comparison with previous experience of face-to-face care (survey questions)

**Outcomes**

- Studies reporting any quantitative measures related to (1) efficiency (eg, service costs and follow-up care), (2) effectiveness (eg, health outcomes), (3) patient safety (eg, misdiagnoses), (4) patient-centeredness (eg, patient satisfaction measures), (5) timeliness (eg, wait times), and (6) equity (eg, disparities in access or outcomes between different patient subgroups)

**Study type**

- Randomized controlled trials, cluster randomized trials, quasi-experimental studies, case-control studies, cohort studies, cross-sectional studies, and cost-effectiveness studies

**Exclusion criteria.**

**Population (and setting)**

- Patients accessing secondary, tertiary, or quaternary care; direct-to-consumer services; care delivered in retail clinics; or care that is not integrated into primary care

**Intervention**

- Consultations involving only asynchronous communication, synchronous web-based messaging, remote patient monitoring, automated services, or interventions for education or administrative purposes; consultations delivered by non–health care professionals or specialist clinicians; or consultations delivered in retail clinics or via direct-to-consumer models

**Comparator**

- No face-to-face comparison group or no indication of comparison with face-to-face care

**Outcomes**

- Studies reporting only qualitative outcomes; studies reporting outcomes that do not fit under any of the Institute of Medicine’s quality framework domains; or studies evaluating only prescribing outcomes, as changes in prescription patterns are not necessarily reflective of the quality of care and are highly context-specific

**Study type**

- Incomplete studies, commentary articles, systematic reviews, interim reports, scoping reviews, case series, case reports, opinion pieces, or trial protocols

### Screening and Data Extraction

Duplicates were automatically excluded using Covidence web-based screening software (Veritas Health Innovation [27]). During the subsequent title, abstract, and full-text screening processes, the decision to exclude a study was made through consensus between the first and second independent reviewers. Iterative meetings were held to discuss any discrepancies that arose. Only when consensus was not able to be reached, a third reviewer, the most senior member of the research team, made the final decision. Cohen κ was used to measure the intercoder agreement in title and abstract screening and full-text screening (0.22 and 0.65, respectively). Disagreements were resolved through discussion with a third investigator. Data extraction was conducted using a standardized data extraction form (Multimedia Appendix 2). Effect sizes such as mean differences, odds ratios, and risk ratios were extracted. Where available, the rates of intervention adherence, follow-up, and survey response were also extracted.

### Risk of Bias Assessment

Risk of bias was assessed using the Mixed Methods Appraisal Tool (version 18) [28] by 2 independent reviewers (Multimedia Appendix 3 [29-58]). Disagreements were resolved through consensus with a third reviewer. A study was considered high risk if it scored “yes” in ≤2 dimensions, moderate risk if it scored “yes” in 3 dimensions, and low risk if it scored “yes” in 4 or all dimensions.

### Data Synthesis

A narrative synthesis of the selected studies was performed. The overall characteristics of the included studies are summarized. Pertinent findings across the studies were mapped onto the IOM framework to comprehensively illustrate the evidence for all the quality of care domains. For each domain, relevant outcome data were compiled into summary tables to facilitate the comparison of results across the studies (Multimedia Appendix 4 [29,30,32-34,36,37,41-45,50,51,53,56], Multimedia Appendix 5 [31,36,42,54,55,57,58], Multimedia Appendix 6 [29,45,46,50,52], Multimedia Appendix 7 [39,40,57], and Multimedia Appendix 8 [35,38,43,47-49]). The key findings for the 6 IOM care quality domains are presented.

## Results

### Search Results

**Figure 1:**
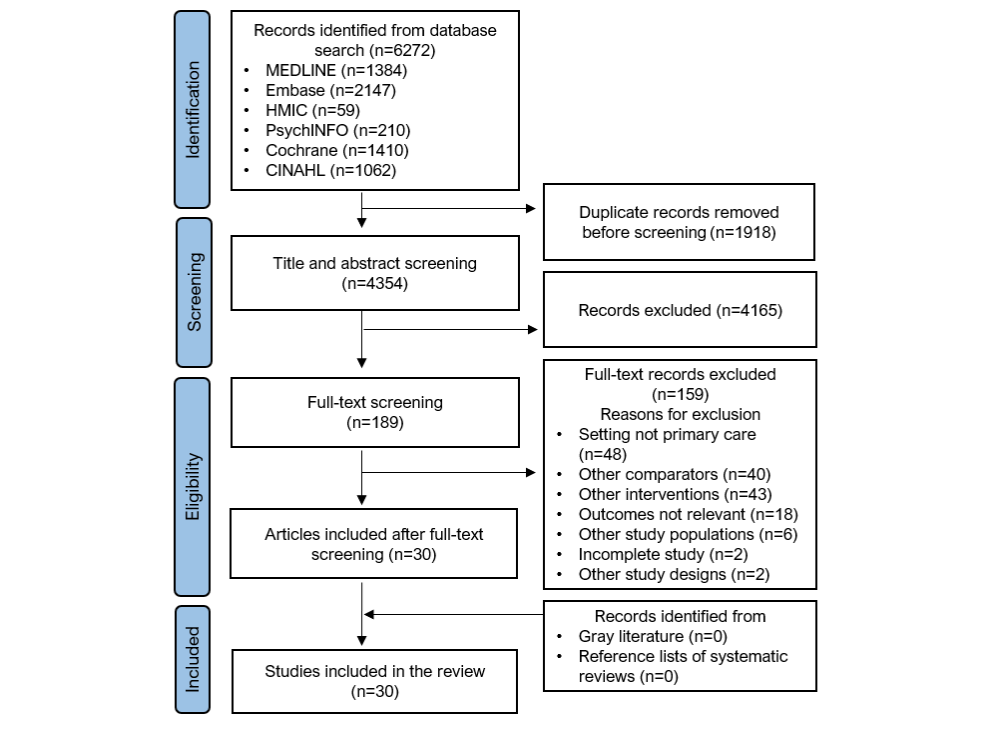
PRISMA (Preferred Reporting Items for Systematic Reviews and Meta-Analyses) flowchart of the included studies.

Our initial searches retrieved a total of 6272 records (Figure 1). After title, abstract, and full-text screening, 30 (0.48%) papers met the inclusion criteria. No relevant records were found from searching gray literature or from the reference lists of relevant articles.

### Description of the Included Studies

The 30 included studies (Table 1) comprised a total of 5,469,333 participants. Sample sizes ranged from 28 [29] to 1,490,734 [30] participants, and publication year ranged from 2017 to 2022. The 30 studies included 14 (47%) retrospective cohorts [30-43], 6 (20%) cross-sectional studies [44-49], 4 (13%) quasi-experimental studies [29,50-52], 3 (10%) randomized controlled trials (RCTs) [53-55], 2 (7%) cohort studies [56,57], and 1 (3%) cluster RCT [58]. Most studies were conducted in the United States (20/30, 67%) [30-34,36-44,46-49,53,58], whereas the rest were conducted in Australia (2/30, 7%) [35,45], Canada (2/30, 7%) [50,52], Kenya (1/30, 3%) [54], the United Kingdom (1/30, 3%) [51], Japan (1/30, 3%) [55], Singapore (1/30, 3%) [29], New Zealand (1/30, 3%) [56], and Sweden (1/30, 3%) [57].

**Table 1:** Characteristics of the included studies.

Study, year	Country	Time period	Study design	Participants (setting)	Sample size (N)	Study design	Consultation description	Risk of bias
Baughman et al [31], 2023	United States	April 2020-September 2021	Retrospective cohort study	Primary care patients aged 26 to 70 years with abnormal BMI scores (<18.5 or >25 kg/m^2^)	287,387 (VC^a^: 1556; blended: 63,489; F2F^b^: 222,333)	Comparison of outcomes among initial visit modalities: VC (Zoom [Zoom Video Communications, Inc] video), blended VC (patients with VC and F2F visits within the time frame), and F2F	Initial BMI screening visits	Moderate
Baughman et al [32], 2022	United States	July 2019-June 2021	Retrospective cohort study	Primary care patients aged 18 to 50 years with a lower back pain diagnosis	20,624 (VC: 5334; F2F: 15,290)	Comparison of outcomes between initial visit modalities: VC (Zoom video) and F2F	Initial primary care consultation	Moderate
Befort et al [58], 2021	United States	February 2016-October 2017	Cluster RCT^c^	Primary care patients aged 20 to 75 (mean age 54.7; SD 11.8) years with BMI scores of 30 to 45 residing in rural locations	1407 (group VC: 466; individual F2F: 473; group F2F: 468)	Practices (n=36) randomly assigned to group VC (telephone conference call), group F2F, and individual F2F	Individual F2F: 15-minute behavioral therapy; group VC and F2F: 60-minute group behavioral therapy (14 patients/group)	Moderate
Bernstein et al [33], 2021	United States	November 2015-March 2019	Retrospective cohort study	Ambulatory care patients aged >60 years with urgent or nonemergent conditions	1088 (VC: 115; F2F: 973)	Comparison of outcomes between index visit modalities: VC (video) and F2F	Visit with physician, including clinical assessment, prescriptions, or referrals if appropriate	Moderate
Chavez et al [34], 2022	United States	April 2019-March 2021	Retrospective cohort study	Patients (mean age 54.1; SD 17.8 y) accessing general care at an academic family medicine practice	57,006^d^ (VC: 7577; F2F: 49,429)	Comparison of outcomes between index visit modalities: VC (video or telephone) and F2F (stratified by prepandemic and pandemic time frames)	Consultation with a physician	High
Dai et al [35], 2022	Australia	March 2020-August 2021	Retrospective cohort study	Geriatric primary care patients (general care) aged >65 years in residential aged care facilities	27,980	Assessment of the associations between sociodemographic characteristics and visit modality: VC (video or telephone) or F2F	GP^e^ consultation	Low
Egede et al [53], 2017	United States	September 2006-October 2012	RCT	Older veterans aged >58 years (mean age 63.9; SD 5.1 y) with depression	241 (VC: 120; F2F: 121)	Comparison of outcomes between consultation modalities: VC (videophone) and F2F	60-minute behavioral activation for depression delivered weekly for 8 weeks	Moderate
Frank et al [36], 2021	United States	March 2019-December 2020	Retrospective cohort study	Patients with mental health conditions at an academic primary care practice aged 4 to 73 (mean age 28.32, SD 15.46) years	173	Comparison of outcomes between 2 waves of consultation modalities: VC (video or telephone; March-December 2020) and F2F (March-December 2019)	30-minute mental health appointments	High
Gordon et al [37], 2017	United States	January 2014-May 2015	Retrospective cohort study	Primary care patients aged <65 years receiving care for acute, nonurgent conditions^f^	18,516 (VC: 4635; F2F: 13,881)	Comparison of outcomes between consultation modalities: VC and F2F	Primary care consultations	Moderate
Govier et al [38], 2022	United States	March 2019-July 2021	Retrospective cohort study	Primary care patients aged >18 years diagnosed with COVID-19 between March and July 2020	11,326 (VC: 1360; F2F: 9966)	Assessment of the associations between sociodemographic characteristics and visit modality: VC or F2F	Primary care consultations	Moderate
Graetz et al [39], 2022	United States	January 2016-May 2018	Retrospective cohort study	Primary care patients of all ages (22.2%>65 y; general care)	1,131,722	Comparison of outcomes among consultation modalities: VC (video), VC (telephone), and F2F	Primary care consultations requested by patients using a web-based portal	Moderate
Haderlein et al [40], 2022	United States	March 2018-October 2021	Retrospective cohort study	Veterans (mean age 48.7, SD 16.4 y) seeking mental health care in an urban VA^g^ primary care mental health integration clinic	2479	Comparison of outcomes between consultation modalities: VC (video or telephone) and F2F	Primary care mental health consultations	Low
Harder et al [54], 2020	Kenya	September 2014-December 2015	RCT	Primary care patients (mean age 38 y) with alcohol use disorders in rural primary health center	300 (VC: 104; F2F: 92; waitlist control: 104)	Comparison of outcomes among consultation modalities: VC (mobile phone call), F2F, and waitlist control	One 30-minute motivational interviewing session	High
Li et al [30], 2022	United States	June 2019-September 2020	Retrospective cohort study	Primary care patients (mean age 39.2, SD 22.2 y; general care)	1,490,734	Comparison of outcomes among different levels of VC use in medical practice: high VC use, medium VC use, and low VC use	Primary care consultations	High
Lovell et al [44], 2021	United States	April 2016-March 2017	Retrospective cohort study	Primary care patients aged <64 years accessing care for low-acuity, urgent conditions^h^	5919 (VC: 1531; F2F: 4388)	Comparison of outcomes between consultation modalities: VC (video call) and F2F	Primary care consultations	High
Manski-Nankervis et al [45], 2022	Australia	October 2020-May 2021	Descriptive cross-sectional study	Primary care patients (mean age 31.8, SD 11.4 y) who completed a videoconference call with a health care professional (general care)	499	Web-based survey on the experience with the most recent VC visit (videoconference) in comparison with past experiences of F2F visit	Primary care consultations	High
McGrail et al [50], 2017	Canada	2013-2014	Quasi-experimental (interrupted time series) study with a cross-sectional survey component	Primary care patients aged >18 years living in British Colombia (general care)	Interrupted time series: 29,267 (VC: 7286; F2F: 21,981); survey: 399	Comparison of outcomes between consultation modalities: VC and F2F; web-based survey on the experience with the most recent VC visit in comparison with past experiences of F2F visit	Primary care consultations	Moderate
Miller et al [51], 2019	United Kingdom	June 2014-May 2017	Quasi-experimental (interrupted time series) study	Primary care patients of all ages at an urban general practice in a socioeconomically deprived area (general care)	27,589 (VC: 9113; F2F: 18,476)	Comparison of outcomes between the 2 study phases: F2F (preintervention phase) and VC (telephone-first phase)	Primary care consultations	Moderate
Mohan et al [46], 2022	United States	April-December 2020	Descriptive cross-sectional study	Primary care patients (mean age 48.7, SD 17.67 y) at an academic medical center (general care)	797	Web-based survey on the experience with the most recent VC visit in comparison with past experiences of F2F visit	Primary care consultations	High
Neufeld et al [52], 2022	Canada	September 2020-February 2021	Quasi-experimental study	Primary care patients aged 18 to 87 years (general care)	66 (VC: 32; F2F: 34)	Comparison of outcomes between consultation modalities: VC (telephone) and F2F	Family physician consultations	Moderate
Nomura et al [55], 2019	Japan	March-June 2018	RCT	Primary care patients (mean age 55, SD 11 y) with nicotine dependence	115 (VC: 58; F2F: 57)	Comparison of outcomes between consultation modalities: VC (internet-based video call) and F2F	5 sessions of smoking cessation counseling over 24 weeks with access to a smoking cessation mobile app and an exhaled CO^i^ checker	Low
Pierce and Stevermer [47], 2023	United States	March-April 2020	Analytical cross-sectional study	Primary care patients (mean age 45 y) at an academic family medicine center (general care)	6984	Assessment of the associations between sociodemographic characteristics and visit modality: VC (audio or video) or F2F	Family medicine consultations	High
Quinton et al [48], 2021	United States	March 2019-March 2021	Analytical cross-sectional study	Patients aged >18 years presenting for ambulatory visits at a rural health care provider (general care)	54,559	Assessment of the associations between patient characteristics and visit modality: VC (audio or video) or F2F	Ambulatory care visits	Moderate
Reed et al [49], 2020	United States	January 2016-May 2018	Analytical cross-sectional study	Primary care patients of all ages at a large integrated health care delivery system (general care)	1,131,722	Assessment of the associations between patient characteristics and visit modality: VC (telephone or video) and F2F	Index primary care consultations (no visits within the past 7 days)	Moderate
Reed et al [41], 2021	United States	January 2016-May 2018	Retrospective cohort study	Primary care patients of all ages (mean age 43, SD 22 y) at a large integrated health care delivery system (general care)	1,131,722	Comparison of outcomes between consultation modalities: VC (telephone or video) and F2F	Index primary care consultations (no visits within the past 7 days)	Moderate
Rene et al [42], 2022	United States	October 2019-May 2020	Retrospective cohort study	Primary care patients aged >18 years with depression, anxiety, or both	338 (VC: 181; F2F: 157)	Comparison of outcomes between consultation modalities: VC (pandemic cohort: April-May 2020) and F2F (prepandemic cohort: October-November 2019)	Initial behavioral health consultation, followed by 30-minute visits for behavioral activation, motivational interviewing, and psychoeducation	Moderate
Ryskina et al [43], 2021	United States	March 2020-May 2020	Retrospective cohort study	Primary care patients aged >65 (mean age 75.1, SD 7.5 y; general care)	17,103 (VC: 10,311; F2F: 6792)	Comparison of outcomes between consultation modalities: VC (telephone or video) and F2F	Primary care consultations	Moderate
Tan et al [29], 2022	Singapore	April 2019-May 2019	Quasi-experimental (prospective and self-controlled) study	Active military servicemen (93% aged 18-24 y) accessing primary care at a military medical center (general care)	28	Comparison of outcomes between consultation modalities: VC (on-premises Zoom video) and F2F (immediately following VC)	Primary care consultations with an assistant present to perform auscultations and physical examinations; a symptom collection app was used before VC, and the collected data were available to the VC physician	Moderate
Ure [56], 2022	New Zealand	May-July 2021	Cohort study	Primary care patients of all ages (25% aged <5 y) at a general practice medical center (general care)	454 (VC: 133; F2F: 321)	Comparison of outcomes between consultation modalities: VC and F2F	Primary care consultations following triage via telephone	Moderate
Wickström et al [57], 2018	Sweden	October 2014-September 2016	Cohort study	Primary care patients (VC: mean age 77, SD 13 y; F2F: mean age 75, SD 14 y) with hard-to-heal ulcers	Healing time study: 1988 (VC: 100; F2F: 1888); waiting time study: 200 (VC: 100; F2F: 100)	Comparison of outcomes between consultation modalities: VC (Skype [Skype Technologies] video) and F2F	Consultations involved ulcer diagnosis and discussion of the treatment strategy	Moderate

^a^VC: virtual consultation.

^b^F2F: face-to-face.

^c^RCT: randomized controlled trial.

^d^Number of index visits (number of participants was not reported).

^e^GP: general practitioner.

^f^Conditions included sinusitis, upper respiratory infection, urinary tract infection, conjunctivitis, bronchitis, pharyngitis, influenza, cough, dermatitis, digestive symptoms, and ear pain.

^g^VA: Veterans Affairs.

^h^Conditions included sinusitis, conjunctivitis, urinary tract infection, upper respiratory infection, influenza or pneumonia, bronchitis, dermatitis or eczema, ear pain, digestive symptoms, and cough.

^i^CO: carbon monoxide.

Studies considered patients with a range of health conditions (ie, mental illnesses [36,40,42,53]; urgent and nonemergent conditions [33,44]; overweight and obesity [31,58]; lower back pain [32]; alcohol use disorders [54]; nicotine dependence [55]; hard-to-heal ulcers [57]; and acute, nonurgent conditions [37]). The remaining studies (17/30, 57%) considered primary care users in general. All consultations were delivered in primary care settings. Of the 30 included studies, 3 (10%) studies specified occurring in rural locations [48,54,58], and 1 (3%) study was conducted in an urban, socioeconomically deprived area [51].

### Summary of the Risk of Bias Assessment

**Figure 2:**
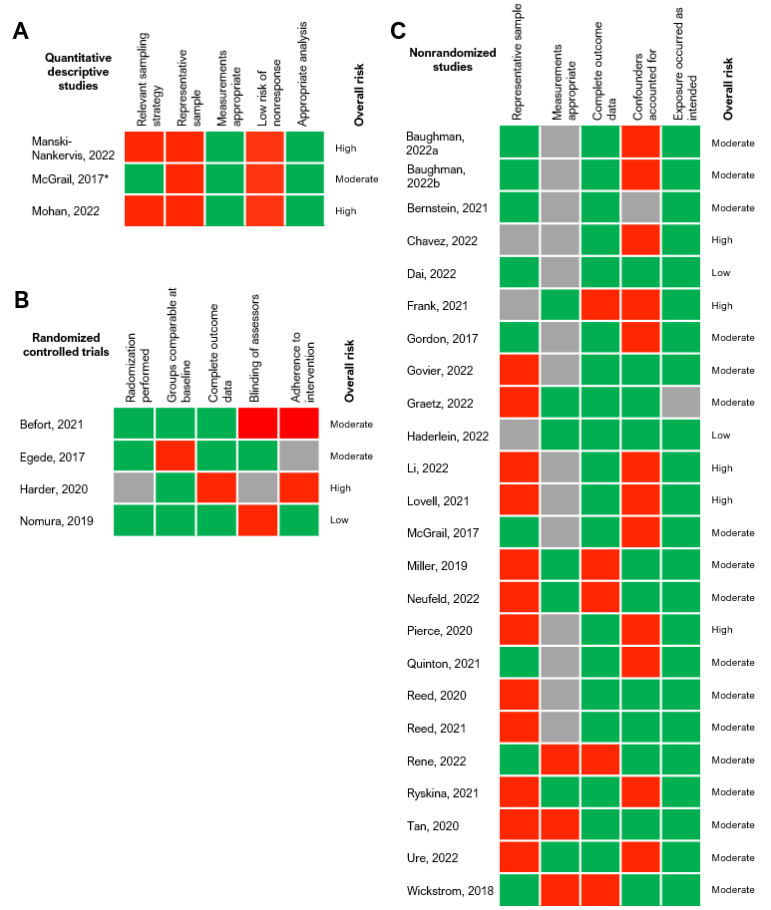
Risk of bias assessments [29-58]. Cells were color coded green for “yes,” gray for “can’t tell,” and red for “no.” *The study by McGrail et al [50] is categorized as both a quantitative descriptive study and nonrandomized trial because of the 2 distinct cross-sectional survey and quasi-experimental components of the study.

Of the 30 included studies, 19 (63%) studies had a moderate risk [29,31-33,37-39,41-43,48-53,56-58], 3 (10%) had a low risk [35,40,55], and 8 (27%) had a high risk of bias [30,34,36,44-47,54] (Figure 2). For quantitative descriptive studies, the main sources of bias were poor sample representativeness and risk of nonresponse bias (ie, potential lower engagement from those with lower digital literacy) [45,46,50]. Main sources of bias for RCTs included issues with blinding [54,55,58], low or unclear adherence to the intervention [53,54,58], lack of details on randomization [54], and differences between groups at baseline [53]. Nonrandomized studies had globally a high risk of bias stemming from the uncertain accuracy of the measurements of exposure and outcome [30-35,37,38,41,44,47-49], unaccounted confounders [30,32,33,43,44,47,48], the overrepresentation of certain subgroups [52], and selection bias [39,41,49].

### Interventions

Of the 30 included studies, 4 (13%) investigated only telephone consultations [51,52,54,58], and 10 (33%) assessed consultations delivered only via videoconference using a range of platforms (ie, Skype [Skype Technologies], Zoom [Zoom Video Communications, Inc], video call, or bespoke telehealth portals) [29,31-33,44-46,53,55,57]. The remaining 16 (53%) of the 30 studies considered consultations via both telephone and videoconference as interventions. In almost all studies, the consultations were the first in an episode of care. Other studies consisted follow-up consultations, including behavioral therapy [42,53,58], motivational interviewing [54], and smoking cessation counseling [55].

### Outcomes

#### Overview

A summary of the main findings of this study can be found in Table 2.

**Table 2:** Summary of the main findings.

Domain of quality	Main findings
Efficiency	Virtual consultations may reduce the rates of hospitalization and follow-up in secondary care but may increase the rate of follow-up in primary care compared with F2F^a^ consultations. Virtual care may lead to lower overall patient spending and be more time-efficient than F2F care.
Effectiveness	Treatment delivered virtually is as effective in improving clinical outcomes as F2F care, particularly for psychological or behavior-related conditions treated in primary care.
Safety	Virtual consultations conducted via videoconference may have similar diagnostic accuracy to F2F for most conditions. There is a lack of studies investigating other aspects of safety, such as medication safety incidents, highlighting an important gap in knowledge.
Patient-centeredness	Patients indicate that virtual consultations are more convenient than and are of similar value and quality to F2F consultations, although findings may be at risk of bias. Virtual consultations may reduce patients’ perceived autonomy support compared with F2F care.
Timeliness	Virtual appointments may have lower wait times than F2F appointments.
Equity	Women are more likely to use virtual consultations than men. The use of virtual care declines with increasing age. The impacts of SES^b^, location of residence, and ethnicity vary considerably between studies and are likely influenced by numerous contextual factors.

^a^F2F: face-to-face.

^b^SES: socioeconomic status.

#### Efficiency

Of the 30 included studies, 16 (53%) evaluated outcomes related to efficiency [29,30,32-34,36,37,41-45,50,51,53,56], including rates of follow-up visits and hospitalizations, patient costs, and appointment characteristics (ie, length, attendance, cancelation, and no-shows; Multimedia Appendix 4). Of these 16 studies, 11 (69%) found a positive impact (n=6, 55%) or no impact (n=5, 45%) on efficiency in at least half of the outcomes extracted [29,32,36,37,41,43-45,50,51,53]. Of the 8 studies comparing rates of follow-up visits in primary care, 5 (63%) found that virtual consultations resulted in a greater need for additional care [33,34,41,44,56], whereas 3 (38%) found no differences [37,50,51].

Out of 7 studies evaluating the rates of follow-up consultations in secondary or tertiary care, 3 (43%) found no changes in the rates of emergency department follow-up visits or hospitalizations [41,44,51]; 3 (43%) other studies [37,43,50] found a significant reduction in follow-up visits after a virtual consultation (including emergency department visits [37], hospitalizations [37,43], and ambulatory care sensitive condition visits [43]); and only 1 (14%) study found that high use of virtual consultations was associated with more annual ambulatory care sensitive condition visits compared with low use of virtual consultations [30].

In terms of appointment characteristics, one study found that virtual consultations led to a higher number of appointments attended and fewer cancelations [36]. However, another study reported lower rates of attendance and increased cancelations in the context of mental health appointments that took place remotely during the pandemic [42]. One study found that the use of virtual consultation and a symptom checker app resulted in a shorter appointment duration when compared with F2F care [29]. Finally, 4 (80%) out of the 5 papers assessing the impact on patient costs demonstrated a reduction in the costs associated with virtual consultations compared with F2F visits [37,44,45,50].

#### Effectiveness

Among the 30 included studies, 7 (23%) assessed the effectiveness of virtual consultations (Multimedia Appendix 5). Of these 7 studies, 6 (86%) found a noninferior impact on effectiveness for at least half of the outcomes [36,42,54,55,57,58]. Furthermore, 2 studies investigating the effectiveness of virtual mental health care in improving anxiety and depression symptoms also demonstrated its noninferiority [42] or superiority [36] to F2F care. Virtual consultations were found to be more effective for the care of hard-to-heal ulcers [57] and as effective as F2F care for reducing alcohol consumption [54], abstinence from smoking [55], and weight management [58].

#### Safety

From the total 30 studies, only 1 (3%) small study (with 28 participants) considered outcomes related to the safety of care, finding an overall diagnostic agreement rate of 92% between virtual and F2F assessments [29]. The agreement rate was 100% for headache, gastroenteritis, and conjunctivitis but lower for dermatological conditions and upper respiratory tract infections (87.5% and 93.3%, respectively) [29].

#### Patient-Centeredness

Of the 5 studies assessing the impact on patient-centeredness, 4 (80%) indicated that virtual care had a positive or equivalent effect compared with F2F care (Multimedia Appendix 6). One (20%) of the studies found that those seen virtually reported lower perceived autonomy support [52]. Three (60%) studies asked patients to compare their recent virtual consultations with past experiences of F2F care [45,46,50], with most respondents agreeing that virtual care was “as good” (84%) [45] and “as thorough” (79%) [50] as F2F care, more convenient (91%) than F2F care, and of equal value to or better value than (67%) F2F care [46]. In another study, most patients (39.9%) had no preference regarding the consultation modality [29].

#### Timeliness

Of the 3 studies evaluating the impact on timeliness, 2 (67%) found an improvement when consultations were delivered virtually (Multimedia Appendix 7). Graetz et al [39] reported that both video and telephone consultations were more likely to occur within 1 day of scheduling. Similarly, a study at a wound healing clinic found that virtual consultations took place significantly sooner after referral [57]. By contrast, a study at a primary care mental health clinic reported that patients who were initially assessed virtually were less likely to receive same-day mental health care [40].

#### Equity

Out of the 30 studies, 6 (20%) assessed the impact on the equity of care through stratification of the use of services based on sex, age, ethnicity, socioeconomic status (SES), and rural or urban residence (Multimedia Appendix 8). Of the 5 studies comparing service use by sex, 2 (40%) studies found that women were more likely to use virtual care than men [47,49]. Regarding age, 2 (n=4, 50%) studies reported that the likelihood of having virtual consultations decreased with increasing age [35,49]. Interestingly, 1 (n=4, 25%) study performed in the first month of the pandemic found that those aged >65 years were more likely to have virtual consultations than those aged 18 to 44 years [47].

Lower use of virtual consultations was reported to be associated with both lower [48,49] and higher SES [35,47]. Notably, 2 (n=5, 40%) studies found that those of lower SES were less likely to have video consultations than they were to have telephone consultations [47,49].

Findings regarding the impact of ethnicity on the use of virtual care were also mixed. Among the 5 studies investigating this, 2 (40%) suggested that Black patients were less likely to use virtual consultations than White patients [47,48], whereas 3 (60%) other studies found the opposite effect [38,43,49]. Asian patients residing in the United States were more likely to use video consultations than White patients but were slightly less likely to use telephone consultations [49].

Finally, a US study reported that patients living in rural areas had lower virtual care use [47], whereas an Australian study found the inverse effect [35].

## Discussion

### Summary of the Main Findings

Our findings suggest that virtual consultations are equally effective to or more effective than F2F care for the management of conditions, including mental illness, excessive smoking, and alcohol consumption. Evidence for the impact on clinical safety is extremely limited. Only 5 studies investigated patient-centeredness, and of these, 4 (80%) found positive impacts on some aspects of patient-centeredness; however, a negative impact was noted on patients’ perceived autonomy support (ie, the degree to which people perceive those in positions of authority to be autonomy supportive). Virtual consultations may reduce waiting times, lower patient costs, and reduce rates of follow-up in secondary and tertiary care settings. However, there is evidence that virtual consultations may increase the need for additional general practitioner visits compared with F2F consultations. Evidence regarding equity was considerably mixed. Overall, it appears that virtual care is more likely to be used by younger, female patients, with disparities among other subgroups depending on contextual factors.

### Interpretation of the Findings in the Context of Previous Research

#### Efficiency

The indication that virtual consultations may increase the rate of follow-up visits in primary care is consistent with previous evidence [3,16]. This negative impact on efficiency may to some extent be explained by the time frames during which the studies were conducted. In those occurring shortly after the onset of the pandemic [34,56], when the rapid transition to virtual care was necessary, the increased rate of follow-up may be a consequence of lower clinician or patient confidence in virtual care owing to its initial unfamiliarity [37].

Previous reviews have not specifically assessed the impact of virtual consultations on follow-up at the secondary or tertiary levels of care. This review’s finding that they may reduce or have no impact on follow-up at these higher levels of care might be explained by retrospective study designs precluding adequate adjustment for confounders and by the heterogeneity of the interventions included [33,37,41,43].

The finding that virtual care may be associated with lower patient costs is in line with previous research [3,17]. Although this evidence is most relevant to countries in which patients pay for services out of pocket, it appears that patients accessing publicly funded health systems may also financially benefit from virtual care, mainly because of reductions in travel expenses and time costs from loss of work [3,44,45]. Furthermore, this review and wider evidence indicate that virtual consultations are generally shorter than F2F visits [3,16,29]. The decrease in consultation length reported by Tan et al [29] may be explained by the use of a symptom checker app before the virtual consultation, which could have improved efficiency for clinicians. However, it is so far unclear whether shorter appointments are indeed more cost-effective than longer appointments and whether they allow enough time for patients to discuss more complex matters.

#### Effectiveness

Virtual care seems to be as effective as F2F visits for certain clinical outcomes (ie, depression and anxiety symptoms, alcohol use disorder scores, smoking abstinence rates, and ulcer healing times). Existing reviews have similarly found noninferior outcomes when virtual care was delivered by specialists [3,12], combined with remote patient monitoring [13], or offered in addition to F2F care [15]. However, this study suggests that virtual consultations may be an effective substitute for F2F consultations in primary care settings.

#### Patient-Centeredness

The wider literature has reported similarly positive findings in terms of patient satisfaction across various types of secondary care and remote patient monitoring for diabetes. [18,19]. One possible explanation is that the use of virtual consultations can lead to less time pressure for physicians and more patient-centric consultations. However, the notable positive impact on patient-centeredness (ie, convenience and preference) should be interpreted with caution owing to the significant risk of bias in these studies and the heterogeneity of measures used. Many of the included studies relied on nonvalidated surveys to assess satisfaction. In this review, the only study using validated questionnaires found that virtual care led to lower perceived autonomy support, possibly because of the absence of nonverbal cues and decreased relational competence [52].

#### Timeliness

There is some evidence that opting for virtual care may reduce wait times for initial consultations compared with F2F care [39,57], possibly facilitated by removing the barriers of job flexibility and travel times [39]. Shorter wait times are a key benefit of virtual care perceived by patients [21]. However, the finding that wait times for mental health care were increased following a virtual appointment may reflect the possibility that patients with lower clinical need chose virtual consultations to begin with (and thus their concerns were assessed as less urgent) or indicate logistical issues in transitions of care [40].

#### Equity

Our findings regarding age and sex are in line with previous research [22] and reflect older patients’ lower average digital literacy and access to technology [47]. Evidence concerning the impacts of SES, ethnicity, and location of residence on the use of virtual care was inconsistent across studies, potentially because of differences in populations and study settings. To this end, access to and use of care are highly context-specific and will be shaped by both community- and practice-level features. As engagement and participation in care are generally lowest for socially disadvantaged populations, disentangling the patient characteristics that may exclude them from virtual care services, at the local level, is essential.

#### Safety

Limited conclusions can be drawn regarding the safety of virtual consultations, as only 1 (n=30, 3%) study investigating outcomes in this domain was identified in this review.

### Strengths and Limitations

This study provides insight into the recent changes in the delivery of primary care and the impact of virtual consultations on care quality. The review uses the IOM’s comprehensive quality framework and maps findings to this model, lending a structured approach to the evaluation of the impact of virtual consultations.

This review did not consider pediatric populations, care delivered by non–health care professionals (eg, community health workers), or outcomes related to medication prescriptions. Many of the included studies considered patients who were part of specific subpopulations, such as older veterans [53] or young military servicemen [29], which may limit the generalizability of the findings.

Eligible studies were restricted to those published within the last 5 years; although this time frame was considered reasonable in line with the changing health care landscape, this might have potentially led to some earlier papers being missed. The decision to conduct the literature search only up to June 2022 may have excluded some more recently studies published in the past year. However, this should not substantially impact our review’s findings, given that it is largely in line with the median time of approximately 8 months as reported in other literature reviews [25].

It is important to note that this study was neither specifically designed to evaluate changes after the COVID-19 pandemic nor optimized to detect those differences. In addition, the studies that covered the COVID-19 pandemic were conducted when the pandemic was in its early stages and, therefore, cannot provide additional information on how the pandemic accelerated adoption, or its impact, after the COVID-19 pandemic.

All but one of the included studies were from high-income countries, and most were from the United States, highlighting the lack of research from low- and middle-income countries. Therefore, many of the findings of this review will have limited relevance outside of the United States and certainly will lack generalizability in low-income regions with dissimilar health system financing structures or technological infrastructure. The lack of studies evaluating safety aspects, such as medication safety incidents, demonstrates the gap in knowledge previously highlighted by Gleeson et al [20]. Similarly, current evidence has common limitations of bias introduced through the lack of adjustment for confounders and selection bias toward the inclusion of patients with greater digital literacy or engagement with health care.

Finally, the apparent lack of studies investigating the effectiveness of virtual care for a wider range of morbidities in primary care highlights a key area for further research.

### Implications for Research, Policy, and Practice

Although evidence of improved efficiency is likely to keep driving the implementation of virtual care, it is critical to ensure that the transition to new service delivery models does not pose additional patient harm. However, even within this domain, the results were sometimes broad and unspecific, making it difficult for readers to contextualize the reported findings. Future research could benefit from a more focused approach when defining the contexts in which evaluations were taking place to aid in the generalizability of the insight gained. The apparent lack of studies investigating the safety of virtual consultations highlights a concerning gap in the literature, and future evaluations should focus on the evaluation of diagnostic error and medication safety in this context. Furthermore, health technology assessments investigating the impact on patient-centeredness should capitalize on the use of validated patient-reported measures whenever possible to allow a rigorous comparative approach.

Policy efforts to support improvements in data collection in primary care (ie, consultation type, duration, and quality outcomes) will be critical to developing a strong evidence base capitalizing on real-world data. The mixed findings on the impact on equity highlight the need for investigations at the local level, which will be vital to develop context-specific strategies tailored to community health needs and characteristics. Data collection should adopt an intersectional approach, considering a breadth of patient characteristics, to inform the design of locally appropriate interventions and ensure equitable access to care. Importantly, virtual consultation interventions and access schemes must incorporate participatory approaches in their research and design, encouraging input from marginalized voices and including community knowledge, values, and preferences in decision-making processes.

## Appendix

Multimedia Appendix 1 MEDLINE search strategy and gray literature search.

Multimedia Appendix 2 Data extraction form template.

Multimedia Appendix 3 Mixed Methods Appraisal Tool assessment with justifications for decisions.

Multimedia Appendix 4 The efficiency of virtual versus face-to face consultations.

Multimedia Appendix 5 The effectiveness of virtual versus face-to-face consultations.

Multimedia Appendix 6 The impact of virtual consultations on patient-centeredness.

Multimedia Appendix 7 The timeliness of virtual versus face-to-face consultations.

Multimedia Appendix 8 The impact of virtual consultations on the equity of care.

## Abbreviations

F2F: face-to-face
IOM: Institute of Medicine
PRISMA: Preferred Reporting Items for Systematic Reviews and Meta-Analyses
RCT: randomized controlled trial
SES: socioeconomic status

## Data Availability

The search strategy and extracted data contributing to the narrative synthesis are available as multimedia appendices. Any additional data are available on request.
