# Supplementary material for: The Impact of Virtual Consultations on the Quality of Primary Care: Systematic Review
Source: J Med Internet Res. 2023 Aug 30;25:e48920. doi: 10.2196/48920 (PMC10500356; doi:10.2196/48920)
Supplement: Multimedia Appendix 1 [file jmir_v25i1e48920_app1.docx]

**Appendix 1**. Medline search strategy and grey literature search.

| *Search items* | *Concepts* |
| --- | --- |
| 1. (telemedicine or tele medicine or telehealth or tele health or telecare or tele care or teleconsult* or ((virtual* or remote* or telephon* or phone* or video* or online) adj3 (consult* or appointment*))) 2. telemedicine/ or remote consultation/ 3. 1 or 2 | Virtual consultation |
| 1. (primary care or primary health care or primary healthcare or general practic* or general medical practice or family medicine or family practic* or family physician*) 2. exp General Practice/ 3. Primary Health Care/ 4. 4 or 5 or 6 | Primary care |
| 1. (((experience* or satisfaction) adj4 (patient* or consumer* or client* or survey* or questionnaire*)) or PREM* or patient-reported experience measure* or patient-cent?red* or person-cent?red*) 2. Patient Satisfaction/ 3. 8 or 9 | Patient-centredness |
| 1. Treatment Outcome/ or ((health or clinical* or treatment*) adj3 (outcome* or effective* or efficacy)) | Effectiveness |
| 1. ((patient adj3 (safety or harm)) or misdiagnos* or safety manag* or (accident* adj2 prevent*) or error* or medication reconcil* or near miss*) 2. patient harm/ or patient safety/ or Diagnostic Errors/ 3. 12 or 13 | Safety |
| 1. (efficiency or economic* or cost* or expenditure* or charge* or (number adj3 appointment*) or (number adj3 admission*) or (number adj3 consultation*)) | Efficiency |
| 1. (wait* list* or wait* time* or timeliness) 2. Time-to-Treatment/ or Waiting Lists/ 3. 16 or 17 | Timeliness |
| 1. ((health* or health care or access) adj3 (equity or disparit* or inequit* or inequalit* or equality or gap)) 2. Health Equity/ 3. 19 or 20 | Equity |
| 1. 10 or 11 or 14 or 15 or 18 or 21 2. 3 and 7 and 22 |  |
