# Supplementary material for: The Impact of Virtual Consultations on the Quality of Primary Care: Systematic Review
Source: J Med Internet Res. 2023 Aug 30;25:e48920. doi: 10.2196/48920 (PMC10500356; doi:10.2196/48920)
Supplement: Multimedia Appendix 2 [file jmir_v25i1e48920_app2.docx]

**Appendix 2**. Data extraction form template

| Author, year |  |
| --- | --- |
| Country of publication |  |
| Study type |  |
| Sample size (n (i; c)) |  |
| Date of intervention |  |
| Duration of intervention |  |
| Participants |  |
| Setting |  |
| Source of data |  |
| Study design and comparison |  |
| Retention or adherence rate (n (i; c)) |  |
| Response rate (n (i; c)) |  |
| Consultation description |  |
| Type of technology |  |
| Outcomes assessed |  |
| Key results (mean difference, OR, RRR) |  |
| Adjustment for confounders |  |
| Domain(s) of quality |  |
| Method of recruitment |  |
| Source of funding |  |
| Possible conflicts of interest |  |
