# Supplementary material for: The Impact of Virtual Consultations on the Quality of Primary Care: Systematic Review
Source: J Med Internet Res. 2023 Aug 30;25:e48920. doi: 10.2196/48920 (PMC10500356; doi:10.2196/48920)
Supplement: Multimedia Appendix 3 [file jmir_v25i1e48920_app3.docx]

**Appendix 3**. Mixed Methods Appraisal Tool assessments with justifications for decisions.

| QUANTITATIVE DESCRIPTIVE STUDIES | | | | | | |
| --- | --- | --- | --- | --- | --- | --- |
| Author, year | Is the sampling strategy relevant to address the research question? | Is the sample representative of the target population? | Are the measurements appropriate? | Is the risk of nonresponse bias low? | Is the statistical analysis appropriate to answer the research question? | Comments |
| Manski-Nankervis, 2022 [45] | No | No | Yes | No | Yes | Sampling strategy (online surveys) may have led to a selection bias Sample was not representative of target population (highly educated, young and majority female)  Low response rate likely to introduce bias |
| McGrail, 2017 [50] | Yes | No | Yes | No | Yes | Sampling strategy (online surveys) may have led to a selection bias  Low response rate likely to introduce bias  Survey sample was not representative of entire target population (majority female and married)  No matching for race and ethnicity which may confound results |
| Mohan, 2022 [46] | No | No | Yes | No | Yes | 26.7% response rate may have introduced bias and selected for those with better digital literacy Overrepresentation of participants who were female and from higher educational/income backgrounds  Study only captures those who could attend a remote visit |
| RANDOMISED CONTROLLED TRIALS | | | | | | |
| Author, year | Is randomization appropriately performed? | Are the groups comparable at baseline? | Are there complete outcome data? | Are outcome assessors blinded to the intervention provided? | Did the participants adhere to the assigned intervention? | Comments |
| Befort, 2021 [58] | Yes | Yes | Yes | No | No | No blinding of outcome assessors to the intervention  Session attendance was notably different between the groups from 6 - 24 months |
| Egede, 2017 [53] | Yes | No | Yes | Yes | Can't tell | There were significant differences between groups in health status compared to the previous year (*P* = 0.02) Unclear if there were differences between treatment groups in terms of completion of interventions |
| Harder, 2020 [54] | Can't tell | Yes | No | Can't tell | No | No description of how randomisation was performed23% of those initially randomised did not complete intervention  No mention of assessors being blinded to the interventions. |
| Nomura, 2019 [55] | Yes | Yes | Yes | No | Yes | Outcome assessors were not blinded to intervention assignment |
| NON-RANDOMISED STUDIES | | | | | | |
| Author, year | Are the participants representative of the target population? | Are measurements appropriate regarding both the outcome and intervention (or exposure)? | Are there complete outcome data? | Are the confounders accounted for in the design and analysis? | During the study period, is the intervention administered (or exposure occurred) as intended? | Comments |
| Baughman, 2022a [31] | Yes | Can't tell | Yes | No | Yes | Possible in accuracies in recording visit modality  Considerable variability of eligible follow-up plans which may have taken varying lengths of time to complete  Confounders such as number of visits or type of follow up plan were not accounted for |
| Baughman, 2022b [32] | Yes | Can't tell | Can't tell | No | Can't tell | Possible in accuracies in recording visit modality and identifying patients with red flag complaints  Confounders such as severity of pain not accounted for |
| Bernstein, 2021 [33] | Yes | Can't tell | Yes | Can't tell | Yes | Possible in accuracies in recording visit modality  Possible misclassification of resolved episodes due to 30-day cut off window  Confounders mostly accounted for except for severity of condition |
| Chavez, 2022 [34] | Can't tell | Can't tell | Yes | No | Yes | No data on patient characteristics other than mean age and sex  Possible in accuracies in recording visit modality  Possible misclassification of short interval-follow ups due to 60-day cut off window |
| Dai, 2022 [35] | Yes | Can't tell | Yes | Yes | Yes | Possible in accuracies in recording visit modality  Possible misclassification of pension status |
| Frank, 2021 [36] | Can't tell | Yes | No | No | Yes | Missing data for outcomes on number of psychiatric problems  Confounders not accounted for  Small sample (n = 18) for outcomes on clinical effectiveness  Academic centre may limit generalisability to wider population |
| Gordon, 2017 [37] | Yes | Can't tell | Yes | No | Yes | Possible in accuracies in recording visit modality |
| Govier, 2022 [38] | No | No | Yes | Yes | Yes | Includes only patients who tested positive for COVID-19, may have missed those who did not test and may be biased towards those with better access to testing May not have captured care accessed by patients in a different healthcare system |
| Graetz, 2022 [39] | No | Yes | Yes | Yes | Can't tell | Sample may not be representative of target population as only included appointments booked via online portal  Unclear if the appointments actually occurred by the modality requested by the patient |
| Haderlein, 2022 [40] | Can't tell | Yes | Yes | Yes | Yes | Sample may not be representative of the wider target population |
| Li, 2022 [30] | No | Can't tell | Yes | No | Yes | May not be wholly representative of the target population Potential inaccuracies of claims data  Possible confounders are not accounted for as data is unknown |
| Lovell, 2021 [44] | No | Can't tell | Yes | No | Yes | May not be wholly representative of the target population  Possible confounders not accounted for as data is unknown |
| McGrail, 2017 [50] | Yes | Can't tell | Yes | No | Yes | Sampling strategy (online surveys) may have led to a selection bias Low response rate likely to introduce bias  Survey sample was not representative of entire target population (majority female and married)  No matching for race and ethnicity which may confound results |
| Miller, 2019 [51] | No | Yes | No | Yes | Yes | May not be generalisable to wider population  Did not report any numerical evidence for change in delays to appointment |
| Neufeld, 2022 [52] | No | Yes | No | Yes | Yes | Sample not representative of wider population (middle-to-upper class, majority white and female)  Use of convenience sampling at the physician's discretion may also have led to some selection bias  Survey response rate was 63.5% |
| Pierce, 2020 [47] | No | Can't tell | Yes | No | Yes | Sample may not be representative of wider population  Potential inaccuracies of claims data Possible confounders not accounted for |
| Quinton, 2021 [48] | Yes | Can't tell | Yes | No | Yes | Potential inaccuracies of data  Possible confounders not accounted for |
| Reed, 2020 [49] | No | Can't tell | Yes | Yes | Yes | Sample may not be representative of wider population Potential inaccuracies of data and misclassification of patients in sociodemographic groups (e.g. socioeconomic status was inferred from area level data). |
| Reed, 2021 [41] | No | Can't tell | Yes | Yes | Yes | Sample may not be representative of wider population Potential inaccuracies of data and misclassification of index visits or patients requiring follow-ups due to the 7 day time frame used |
| Rene, 2022 [42] | Yes | No | No | Yes | Yes | Potential inaccuracies of data  Unclear follow-up period |
| Ryskina, 2021 [43] | No | Yes | Yes | No | Yes | Hispanic patients were underrepresented in the sample  Possible confounders not accounted for |
| Tan, 2020 [29] | No | No | Yes | Yes | Yes | Sample very small and not representative of wider military population  Did not use a validated satisfaction questionnaire |
| Ure, 2022 [56] | No | Yes | Yes | No | Yes | Not representative of wider population  Possible confounders not accounted for |
| Wickstrom, 2018 [57] | Yes | No | No | Yes | Yes | Use of different measurement techniques between the study and control groups may have resulted in differences between groups  Low rates of 6 month follow up, especially for the control group |
