# Supplementary material for: The Impact of Virtual Consultations on the Quality of Primary Care: Systematic Review
Source: J Med Internet Res. 2023 Aug 30;25:e48920. doi: 10.2196/48920 (PMC10500356; doi:10.2196/48920)
Supplement: Multimedia Appendix 4 [file jmir_v25i1e48920_app4.docx]

**Appendix 4*.*** The efficiency of virtual vs face-to face (F2F) consultations.

| *Author, year* | *Outcome measure* | *VC mean (95% CI or SD)* | *F2F mean (95% CI or SD)* | *Mean difference or effect size (95% CI and/or P value)* | *Risk of bias* |
| --- | --- | --- | --- | --- | --- |
|  | **a) Rates of follow up and hospitalizations** | | |  |  |
| Bernstein, 2021 [33] | Visit resolution (no follow-up within 30 days) (%) | 84.00 (76.50, 90.90) | 90.70 (87.70, 93.40) |  | Moderate |
|  | Episodes of care required for resolution | 2.50 (2.11, 2.72) | 2.90 (2.01, 2.42) |  |  |
|  | Index visits requiring additional episodes of care (%) | 14.80 | 8.10 |  |  |
| Chavez, 2022 [34] | Short-interval follow-up rate (any visit within 60 days) (%) | 22.80 | 16.80 | (*P* < 0.001) | High |
|  | Visits requiring follow-up within 15 days (%) | 31.34 | 23.44 | (*P* < 0.001) |  |
| Li, 2022 [30] | Rate of ACSC visits^a^ (high practice VC vs low practice VC) |  |  | 2.10 (0.22, 3.97) | High |
|  | Rate of ACSC visits^a^ (medium practice VC vs low practice VC) |  |  | 0.69 (-0.93, 2.21) |  |
| Lovell, 2021 [44] | ED follow-up rate (within 21 days) (%) | 1.80 | 2.60 | 1.49 (0.87, 2.12;*P* > 0.05) | High |
|  | Inpatient follow-up rate (within 21 days) (%) | 0.40 | 0.70 | 1.77 (0.22, 3,32;P > 0.05) |  |
|  | Any visit follow-up rate (within 21 days) (%) | 35.30 | 35.70 | 1.01 (0.94, 1.09:*P* > 0.05) |  |
|  | E&M follow-up rate (within 21 days) (%) | 26.60 | 22.60 | 0.85 (0.77, 0.93;*P* < 0.001) |  |
| McGrail, 2017 [50] | GP follow-up rate (within 30 days) (%) | 1.55 | 1.43 | (*P* = 0.45) | Moderate |
|  | Medical specialist follow-up rate (within 30 days) (%) | 1.72 | 1.62 | (*P* = 0.58) |  |
|  | Surgical specialist follow-up rate (within 30 days) (%) | 1.07 | 2.18 | (*P* < 0.001) |  |
| Gordon, 2017 [37] | Outpatient follow-up rate (within 3 weeks) (%) | 28.09 | 28.10 | (*P* = 0.96) | Moderate |
|  | ED follow-up rate (within 3 weeks) (%) | 1.32 | 1.84 | (*P* = 0.02) |  |
|  | Inpatient hospitalization (within 3 weeks) (%) | 0.15 | 0.37 | (*P* = 0.02) |  |
| Reed, 2021 [41] | Office follow-up rate (within 7 days) (%) (VC = video) | 25.40 (24.70, 26.00) | 24.50 (24.50, 24.60) | (*P* < 0.05) | Moderate |
|  | Office follow-up rate (within 7 days) (%) (VC = telephone) | 26.00 (25.90, 26.20) | 24.50 (24.50, 24.60) | (*P* < 0.05) |  |
|  | ED follow-up rate (within 7 days) (%) (VC = video) | 1.23 (1.06, 1.40) | 1.30 (1.29, 1.32) | (*P* > 0.05) |  |
|  | ED follow-up rate (within 7 days) (%) (VC: telephone) | 1.37 (1.33, 1.41) | 1.30 (1.29, 1.32) | (*P* > 0.05) |  |
|  | Hospitalization rate (within 7 days) (%) (VC = video) | 0.23 (0.14, 0.32) | 0.23 (0.22, 0.24) | (*P* > 0.05) |  |
|  | Hospitalization rate (within 7 days) (%) (VC = telephone) | 0.22 (0.21, 0.24) | 0.23 (0.22, 0.24) | (*P* > 0.05) |  |
| Ure, 2022 [56] | Rate of re-triage (within 7 days) (%) | 14.00 | 7.00 | (P < 0.05) | Moderate |
| Ryskina, 2021 [43] | Odds of ACSC hospitalization (within 14 days) (VC vs F2F) |  |  | 0.78 (0.61, 1.00; *P* = 0.049) | Moderate |
|  | Odds of all-cause hospitalization (within 14 days) (VC vs F2F) |  |  | 0.72 (0.57, 0.90; *P* = 0.004) |  |
| Miller, 2019 [51] | Number of GP visits per patient per year |  |  | (*P* = 0.193) | Moderate |
|  | Number of referrals to hospital per month | 171 | 182 | 11 (*P* = 0.181) |  |
|  | Number of visits to GP out-of-hours per month | 313 | 304 | 9(*P* = 0.56) |  |
|  | Sum of local ED visits per intervention period | 3700 of 66228 | 6771 of 122428 | (*P* = 0.73) |  |
|  | **b) Patient costs** |  |  |  |  |
| Gordon, 2017 [37] | Cost of index visit (USD) | $49 | $109 | $60 (*P* < 0.001) | Moderate |
|  | Average medical costs within 3-week follow-up (USD) | $200 | $288 | (*P* < 0.001) |  |
| Lovell, 2021 [44] | Cost of index visit (USD) | $45 | $114 | 2.54 (2.46, 2.62;*P* < 0.001) | High |
|  | Pharmacy costs within 21 days of index (USD) | $111 | $117 | 1.06 (0.82, 1.29; *P* > 0.05) |  |
|  | Follow-up costs within 21 days (USD) | $288 | $490 | 1.70 (1.04, 2.36;*P* = 0.038) |  |
|  | Average total costs (USD) | $429 | $707 | 1.65 (1.26, 2.04;*P* < 0.001) |  |
| Egede, 2017 [53] | Inpatient cost trajectories over time^b^ (VC vs F2F) |  |  | 0.17 (*P* = 0.845) | Moderate |
|  | Outpatient cost trajectories over time^b^ (VC vs F2F) |  |  | −0.07 (*P* = 0.071) |  |
|  | Pharmacy cost trajectories over time^b^ (VC vs F2F) |  |  | 0.01 (*P* = 0.805) |  |
| Manski-Nankervis, 2022 [45] | Mean total cost saving^c^ from VC vs F2F (AUD) |  |  | $61.36 ($53.24, $69.48) | High |
| McGrail, 2017 [50] | Trend in cost of primary care services^d^ (CAD) (VC vs F2F) |  |  | -$3.79 (*P* = 0.01) | Moderate |
|  | **c) Appointment characteristics** | |  |  |  |
| Frank, 2021 [36] | Number of appointments attended | 2.17 (4.46) | 1.19 (2.08) | (*P* = 0.002) | High |
|  | Number of appointment cancellations | 0.14 (0.49) | 0.53 (1.03) | (*P* < 0.001) |  |
| Rene, 2022 [42] | Number of appointment cancellations | 0.45 (0.81) | 0.36 (0.76) | (*P* = 0.003) | Moderate |
|  | Number of appointment no-shows | 0.38 (0.67) | 0.25 (0.54) | (*P* = 0.26) |  |
|  | Number of appointments attended | 2.15 (2.24) | 3.32 (1.49) | (*P* < 0.001) |  |
| Tan, 2020 [29] | Consultation length | 6 min 19 sec | 8 min 34 sec | (*P* = 0.048) | Moderate |
| Baughman, 2022b [32] | Patients receiving imaging within 28 days (%) | 11.20 | 16.32 | 5.12(*P* < 0.01) | Moderate |

ACSC, ambulatory care-sensitive conditions; CI, confidence intervals; ED, emergency department; E&M, evaluation and management; F2F, face-to-face; GP, general practitioner; SD, standard deviation; VC, virtual consultation

^a^ Visits per 1000 patients per year

^b^ Including the 8-week intervention period, and the two years prior- and post-intervention

^c^ Including paid work, unpaid time and travel costs

^d^ Average change in spending per quarter over 3 years
