# Supplementary material for: The Impact of Virtual Consultations on the Quality of Primary Care: Systematic Review
Source: J Med Internet Res. 2023 Aug 30;25:e48920. doi: 10.2196/48920 (PMC10500356; doi:10.2196/48920)
Supplement: Multimedia Appendix 5 [file jmir_v25i1e48920_app5.docx]

**Appendix 5**. The effectiveness of virtual vs face-to-face (F2F) consultations.

| *Author, year* | *Outcome measure* | *VC mean (95% CI or SD)* | *F2F mean (95% CI or SD)* | *Mean difference (95% CI and/or P value)* | *Risk of bias* |
| --- | --- | --- | --- | --- | --- |
| Baughman, 2022a [31] | Patients with completed weight management plan at 1.5 years (%) | 7.90 | 12.19 | 4.29(2.84, 5.54: *P* < 0.001) | Moderate |
|  | Patients with completed weight management plan at 3 months (%) | 1.80 | 6.59 | 4.79(3.99, 5.35;P < 0.001) |  |
|  | Patients with completed weight management plan at 1.5 years (%) (blended VC vs F2F) | 24.82 | 12.19 | 12.65%(12.29, 13.01;P < 0.001) |  |
|  | Patients with completed weight management plan at 3 months (%) (blended VC vs F2F) | 17.75 | 6.59 | 11.16 (10.85, 11.48;P < 0.001) |  |
| Befort, 2021 [58] | Weight loss (kgs) at 24 months (VC vs group F2F) | –3.90 (–5.00, –2.90) | –4.40 (–5.50, –3.40) | –0.50 ( –1.90, 0.90;*P* = 0.48) | Moderate |
|  | Weight loss (kgs) at 24 months (VC vs individual F2F) | –3.90 (–5.00, –2.90) | –2.60 (–3.60, –1.50) | –1.40 (–3.00, 0.30;*P* = .06) |  |
| Frank, 2021 [36] | CGI-S scores at end of 8-month wave | 3.33 (0.97) | 3.61(0.70) | (*P* = 0.02) | High |
|  | CGI-I scores at end of 8-month wave | 2.44 (0.51) | 3.06 (0.87) | (*P* = 0.002) |  |
| Rene, 2022 [42] | Change in PHQ-9 score | -2.90 | -2.80 | (*P* > 0.05) | Moderate |
|  | Change in GAD-7 score | -3.10 | -2.30 | (*P* > 0.05 |  |
| Harder, 2020 [54] | AUDIT-C scores at 1 month follow-up |  |  | 0.20(-0.60, 1.00;*P* = 0.63) | High |
|  | AUDIT-C scores at 6 months follow-up |  |  | 0.44(-0.47, 1.36; *P* = 0.34) |  |
| Nomura, 2019 [55] | CAR (%) from weeks 9-12 | 81.00 (71.00, 91.00) | 78.90 (68.00, 89.00) | 2.10 (–12.80, 17.00) | Low |
|  | CAR (%) from weeks 9 -24 | 74.10 (63.00, 85.00) | 71.90 (60.00, 84.00) | 2.20 (–14.00, 18.40) |  |
| Wickstrom, 2018 [57] | Number of days between consultation and complete  ulcer healing | 78 (40, 78) | 118 (75, 89) | (*P* < 0.001) | Moderate |

AUDIT-C, Alcohol Use Disorders Identification Test – Consumption; CAR, continuous abstinence rate; CGI-I, Clinical Global Impressions – Improvement; CGI-S, Clinical Global Impressions – Severity; CI, confidence intervals; F2F, face-to-face; GAD-7, Generalised Anxiety Disorder-7; kgs, kilograms; PHQ-9, Patient Health Questionnaire-9; SD, standard deviation; VC, virtual consultation
