# Supplementary material for: The Impact of Virtual Consultations on the Quality of Primary Care: Systematic Review
Source: J Med Internet Res. 2023 Aug 30;25:e48920. doi: 10.2196/48920 (PMC10500356; doi:10.2196/48920)
Supplement: Multimedia Appendix 6 [file jmir_v25i1e48920_app6.docx]

**Appendix 6.** The impact of virtual consultations on patient-centredness.

| *Author, year* | *Outcome measure* | *VC mean (SE)* | *F2F mean (SE)* | *Mean difference (P value)* | *Risk of bias* |
| --- | --- | --- | --- | --- | --- |
| Neufeld, 2022 [52] | Patient perceived autonomy support^a^ | 5.75 (0.17) | 6.28 (0.16) | (*P* = 0.032) | Moderate |
|  | **Survey question responses** | | |  |  |
|  |  | ***Agree/ Better*** | ***Neutral/ Equal*** | ***Disagree/ Worse*** | ***Risk of bias*** |
| Manski-Nankervis, 2022 [45] | VC visit was as good as F2F (%) | 84.00 |  | 11.40 | High |
| McGrail, 2017 [50] | VC visit was as thorough as F2F (%) | 79.00 |  | 21.00 | Moderate |
| Mohan, 2022 [46] | Convenience of VC compared to F2F (%) | 91.00 | 4.00 | 4.00 | High |
|  | Value of VC compared to F2F (%) | 37.00 | 30.00 | 33.00 |  |
| Tan, 2020 [29] | F2F visits provide better quality care than VC (%) | 32.00 | 50.10 | 17.90 | Moderate |
|  | Prefer F2F visits over VC in the future (%) | 28.60 | 39.90 | 32.10 |  |

F2F, face-to-face; SE, standard error; RC, remote consultation

^a^ Measured using the Healthcare Climate and Basic Need Satisfaction in Relationships questionnaires
