# Supplementary material for: The Impact of Virtual Consultations on the Quality of Primary Care: Systematic Review
Source: J Med Internet Res. 2023 Aug 30;25:e48920. doi: 10.2196/48920 (PMC10500356; doi:10.2196/48920)
Supplement: Multimedia Appendix 7 [file jmir_v25i1e48920_app7.docx]

**Appendix 7.** The timeliness of virtual vs face-to-face consultations.

| *Author, year* | *Outcome measure* | *VC mean (95% CI or SD)* | *F2F mean (95% CI or SD)* | *Mean difference (P value)* | *Risk of bias* |
| --- | --- | --- | --- | --- | --- |
| Graetz, 2022 [39] | Days between scheduling and visit (VC = telephone) | 1.80 | 3.50 | 1.70 | Moderate |
|  | Days between scheduling and visit (VC = video) | 2.30 | 3.50 | 1.20 |  |
|  | Visits occurring within 1 day of scheduling (%) (VC: telephone) | 66.60 (66.40, 66.80) | 46.50 (46.40, 46.60) |  |  |
|  | Visits occurring within 1 day of scheduling (%) (VC: video) | 56.60 (55.90, 57.30) | 46.50 (46.40, 46.60) |  |  |
| Haderlein, 2022 [40] | Patients receiving same-day mental health care (%) | 19.70 | 36.00 | (*P* < 0.01) | Low |
| Wickstrom, 2018 [57] | Number of days between referral and consultation | 25.00 | 43.00 | (*P* = 0.017) | Moderate |

CI, confidence intervals; F2F, face-to-face; SD, standard deviation; VC, virtual consultation
