# Supplementary material for: The Impact of Virtual Consultations on the Quality of Primary Care: Systematic Review
Source: J Med Internet Res. 2023 Aug 30;25:e48920. doi: 10.2196/48920 (PMC10500356; doi:10.2196/48920)
Supplement: Multimedia Appendix 8 [file jmir_v25i1e48920_app8.docx]

**Appendix 8**. The impact of virtual consultations on equity of care.

| *Author, year* | *Outcome measure* | *Mean difference (%), OR, or RRR* | *95% CI and/or P value* | *Risk of bias* |
| --- | --- | --- | --- | --- |
|  | **a) Use of VC vs F2F** |  |  |  |
| Dai, 2022 [35] | Male vs female | 1.02 | 1.0, 1.04 | Low |
|  | 70-74 vs 65-69 | 0.96 | 0.9, 1.03 |  |
|  | 75-79 vs 64-69 | 0.87 | 0.82, 0.93* |  |
|  | 80-84 vs 65-69 | 0.86 | 0.81, 0.91* |  |
|  | 85+ vs 64-69 | 0.70 | 0.66, 0.74* |  |
|  | Pension holders^a^ vs not on pension | 1.14 | 1.10, 1.17* |  |
|  | Rural vs urban | 1.72 | 1.57, 1.90* |  |
| Pierce, 2020 [47] | Female vs male | 1.15 | 1.04, 1.26* | High |
|  | <18 vs 18-44 | 0.35 | 0.29, 0.41* |  |
|  | 45-65 vs 18-44 | 1.08 | 0.97, 1.21 |  |
|  | >65 vs 18-44 | 1.21 | 1.05, 1.40* |  |
|  | Black vs white | 0.65 | 0.56, 0.75* |  |
|  | Other race vs white | 0.64 | 0.50, 0.82* |  |
|  | Hispanic vs non-Hispanic | 0.87 | 0.62, 1.22 |  |
|  | Self-pay vs insurance | 1.26 | 1.04, 1.52* |  |
|  | Medicaid vs insurance | 1.29 | 1.04, 1.61* |  |
|  | Medicare vs insurance | 1.37 | 1.18, 1.60* |  |
|  | Rural vs urban | 0.81 | 0.74, 0.90* |  |
| Ryskina, 2021 [43] | Female vs male | 1.11 | 0.99, 1.59 | Moderate |
|  | 75-84 vs 65-74 | 1.1 | 0.97, 1.24 |  |
|  | >84 vs 65-74 | 1.18 | 1.0, 1.41 |  |
|  | Black vs white | 1.30 | 1.14, 1.47* |  |
|  | Asian vs white | 0.73 | 0.47, 1.12 |  |
|  | **b) Use of VC vs no care** |  |  |  |
| Govier, 2022 [38] | Non-Hispanic Black vs non-Hispanic white | + 3.30 | *P* < 0.05* | Moderate |
|  | Non-Hispanic Asian vs non-Hispanic white | + 0.30 | *P* > 0.05 |  |
|  | Non-Hispanic NH/PI vs non-Hispanic white | + 0.60 | *P* > 0.05 |  |
|  | Non-Hispanic other vs non-Hispanic white | − 2.53 | *P* < 0.05* |  |
|  | Hispanic/Latino vs non-Hispanic white | − 2.11 | *P* < 0.01* |  |
|  | SES Theme^b^ | + 0.03 | *P* > 0.05 |  |
|  | Minority Status/Language Theme^b^ | − 0.83 | *P* > 0.05 |  |
| Quinton, 2021 [48] | Female vs male | 0.93 | 0.80, 1.08 | Moderate |
|  | Black vs white | 0.54 | 0.42, 0.70; *P* < 0.05* |  |
|  | Other vs white | 0.86 | 0.44, 1.67 |  |
|  | Self-pay vs commercial | 0.73 | 0.55, 0.96; *P* < 0.05* |  |
|  | Medicaid vs commercial | 0.44 | 0.31, 0.61; *P* < 0.05* |  |
|  | Medicare vs commercial | 0.70 | 0.55, 0.89; *P* < 0.05* |  |
|  | 80-100% broadband access vs 0-20% | 2.24 | 1.70, 2.95; *P* < 0.05* |  |
|  | 60-80% broadband access vs 0-20% | 1.10 | 0.86, 1.41; *P* < 0.05* |  |
|  | 40-60% broadband access vs 0-20% | 1.07 | 0.54, 2.10; *P* < 0.05* |  |
|  | 20-40% broadband access vs 0-20% | 1.09 | 0.86, 1.37 |  |
|  | Partially rural vs rural | 2.64 | 2.25, 3.11; *P* < 0.05* |  |
|  | Non-rural vs rural | 0.69 | 0.26, 1.88 |  |
|  | **c) Use of video vs F2F** |  |  |  |
| Reed, 2020 [49] | Male vs female | 0.93 | 0.90, 0.96* | Moderate |
|  | <18 vs 18-44 | 1.00 | 0.91, 1.09 |  |
|  | 45-64 vs 18-44 | 0.61 | 0.58, 0.63* |  |
|  | >65 vs 18-44 | 0.24 | 0.22, 0.26* |  |
|  | Black vs white | 1.62 | 1.52, 1.73* |  |
|  | Hispanic vs white | 0.92 | 0.88, 0.97* |  |
|  | Asian vs white | 1.26 | 1.22, 1.32* |  |
|  | Low SES neighbourhood vs high | 0.93 | 0.89, 0.97* |  |
|  | **d) Use of telephone vs F2F** |  |  |  |
| Reed, 2020 [49] | Male vs female | 0.80 | 0.79, 0.81* | Moderate |
|  | <18 vs 18-44 | 0.42 | 0.40, 0.43* |  |
|  | 45-64 vs 18-44 | 0.80 | 0.70, 0.81* |  |
|  | >65 vs 18-44 | 0.55 | 0.54, 0.57* |  |
|  | Black vs white | 1.28 | 1.25, 1.31* |  |
|  | Hispanic vs white | 1.00 | 0.98, 1.01 |  |
|  | Asian vs white | 0.96 | 0.94, 0.97* |  |
|  | Low SES neighbourhood vs high | 1.01 | 1.00, 1.03 |  |
|  | **e) Use of video vs telephone** |  |  |  |
| Dai, 2022 [35] | Male vs female | 0.94 | 0.86, 1.02 | Low |
|  | 70-74 vs 65-69 | 0.80 | 0.61, 1.03 |  |
|  | 75-79 vs 64-69 | 1.13 | 0.89, 1.44 |  |
|  | 80-84 vs 65-69 | 1.03 | 0.82, 1.29 |  |
|  | 85+ vs 64-69 | 1.20 | 0.97, 1.49 |  |
|  | Pension holders^a^ vs not on pension | 1.01 | 0.90, 1.13 |  |
|  | Rural vs urban | 0.41 | 0.29, 0.57* |  |
| Pierce, 2020 [47] | Female vs male | 1.08 | 0.91, 1.27 | High |
|  | <18 vs 18-44 | 0.92 | 0.59, 1.45 |  |
|  | 45-65 vs 18-44 | 0.51 | 0.41, 0.62* |  |
|  | >65 vs 18-44 | 0.27 | 0.21, 0.33* |  |
|  | Black vs white | 0.72 | 0.55, 0.93* |  |
|  | Other race vs white | 0.96 | 0.48, 1.82 |  |
|  | Hispanic vs non-Hispanic | 0.93 | 0.48, 1.82 |  |
|  | Self-pay vs insurance | 0.68 | 0.49, 0.95* |  |
|  | Medicaid vs insurance | 0.36 | 0.26, 0.51* |  |
|  | Medicare vs insurance | 0.79 | 0.64, 0.99* |  |
|  | Rural vs urban | 1.36 | 1.14, 1.61* |  |

CI, confidence intervals; F2F, face-to-face; NH/PI, Native Hawaiian/ Pacific Islander; OR, odds ratio; RRR, relative risk ratio; SES, socioeconomic status; VC, virtual consultation

* Statistically significant findings

^a^ Pension holder status was used as a proxy indicator of lower SES

^b^ Reference category indicates those less vulnerable for the respective theme, defined using the Social Vulnerability Index
